# Osimertinib in advanced EGFR-mutant lung adenocarcinoma with asymptomatic brain metastases: an open-label, 3-arm, phase II pilot study

**DOI:** 10.1093/noajnl/vdab188

**Published:** 2021-12-27

**Authors:** Nir Peled, Waleed Kian, Edna Inbar, Iris M Goldstein, Melanie Zemel, Ofer Rotem, Anna B Rozenblum, Hovav Nechushtan, Elizabeth Dudnik, Daniel Levin, Alona Zer, Shoshana Keren-Rosenberg, Shlomit Yust-Katz, Vered Fuchs, Areen A Remilah, Ilan Shelef, Laila C Roisman

**Affiliations:** 1 Department of Oncology, The Institute of Oncology, Shaare Zedek Medical Center, Jerusalem, Israel; 2 Department of Oncology, The Legacy Heritage Center & Dr. Larry Norton Institute, Soroka Medical Center & Ben-Gurion University of the Negev, Be’er Sheva, Israel; 3 Department of Diagnostic Imaging, Rabin Medical Center, Davidoff Cancer Center, Petach Tikva, Israel; 4 Department of Diagnostic Imaging, The Legacy Heritage Center & Dr. Larry Norton Institute, Soroka Medical Center & Ben-Gurion University of the Negev, Be’er Sheva, Israel; 5 Department of Oncology, Rabin Medical Center, Davidoff Cancer Center, Petach Tikva, Israel; 6 Department of oncology, Hadassah Medical Center, Jerusalem, Israel; 7 Department of Oncology, Lin Medical Center, Haifa, Israel; 8 Department of Diagnostic Imaging, Diagnostic Imaging Institute, Soroka University Medical Center, Be’er-Sheba, Israel

**Keywords:** brain metastases, EGFR, LUAD, osimertinib, Thr790Met

## Abstract

**Background:**

Osimertinib is selective for both epidermal growth factor receptor (EGFR)-tyrosine-kinase inhibitor (TKI) sensitizing and Thr790Met mutations. While intracranial activity of osimertinib is documented in larger trials, a prospective study focusing exclusively on patients with asymptomatic brain metastases has not been reported.

**Methods:**

In this nonrandomized, phase II, open-label, 3-arm prospective proof-of-concept pilot study, 48 patients with metastatic EGFR-mutant lung adenocarcinoma (LUAD) received osimertinib 80 mg daily. Patients were either treatment naive (arm A = 20) or previously treated with an EGFR-TKI and Thr790Met positive (arm B = 18) or negative (arm C = 10). In cases of isolated intracranial progression, osimertinib dose was escalated (160 mg). The primary endpoints were intracranial objective response rate (iORR) and intracranial disease control rate (iDCR). The secondary endpoint was intracranial progression-free survival (iPFS). This study is registered at Clinicaltrials.gov, NCT02736513.

**Results:**

The iORRs were 84.2%, 66.7%, and 50% and the iDCRs were 94.7%, 94.4%, and 80% in arms A, B, and C, respectively. The median iPFS was 11.8 months (95% CI 7.7 to NA), 7.6 months (95% CI 5.3 to NA), and 6.3 months (95% CI 3.9 to NA) in arms A, B, and C, respectively. Following dose escalation, pooled iORR was 54% (arm A = 5, arm B = 4, arm C = 2). Adverse events were similar to those in previously published literature.

**Conclusion:**

Osimertinib demonstrated high efficacy on brain metastases. All trial arms displayed a significant decrease in the number and diameter of target lesions. These findings indicate that osimertinib is effective for Thr790Met-positive and -negative LUAD patients with asymptomatic brain metastases. Therefore, osimertinib should be considered a viable option for EGFR-mutant patients with brain involvement regardless of their Thr790Met mutation status.

**Key Points**

- Osimertinib is effective in treatment naive (A), Thr790Met-positive (B), and -negative (C) LUAD.
- In LUAD patients with asymptomatic brain metastases, the iORR was 84.2%, 66.7%, and 50% in A, B, and C, respectively.
- The iDCR was 94.7%, 94.4%, and 80% in arms A, B, and C, respectively.

**Importance of the Study**

Osimertinib is selective for both EGFR-TKI sensitizing and Thr790Met mutations. While intracranial activity of osimertinib has been observed in larger trials, a prospective study focusing exclusively on patients with brain metastases has not yet been reported. This study involved 48 patients with asymptomatic brain metastases. The iORRs were 84.2%, 66.7%, and 50% and the iDCRs were 94.7%, 94.4%, and 80% in arms A, B, and C, respectively. The median iPFS was 11.8 months (95% CI 7.7 to NA), 7.6 months (95% CI 5.3 to NA), and 6.3 months (95% CI 3.9 to NA) in arms A, B, and C, respectively. Following dose escalation in 11 patients, pooled iORR was 54% (arm A = 5, arm B = 4, arm C = 2). A significant decrease in the number and diameter of target lesions was found in all arms. Therefore, we suggest osimertinib be considered as a treatment in EGFR-mutant patients with brain involvement regardless of their Thr790Met mutation status.

First- and second-generation EGFR-tyrosine-kinase inhibitors (TKIs) revolutionized the treatment paradigm of advanced lung adenocarcinoma (LUAD). They became the standard of care for EGFR-mutant lung cancer. Unfortunately, acquired resistance commonly develops after 9–13 months.^1^ The main resistance mechanisms develop through *EGFR* gene mutations, including Thr790Met. Less common mechanisms include ERBB2 amplification, transformation to small-cell lung cancer, mesenchymal-epithelial transition (MET) amplification, and others.^2–4^

Osimertinib, an irreversible, third-generation EGFR-TKI, targets the Thr790Met resistance mutation as well as another common EGFR sensitizing mutations.^5^ In a phase III trial (AURA3), osimertinib demonstrated superior outcomes compared with platinum-based doublet chemotherapy.^6^ Currently, it is the only targeted drug approved by the Food and Drug Administration (FDA) for treatment of LUAD with acquired Thr790Met EGFR mutations, following progression on erlotinib, gefitinib, or afatinib.^7^

In the phase III FLAURA trail, comparing osimertinib to first-generation EGFR-TKIs in advanced EGFR-mutant LUAD, objective response rates (ORRs) were similar, and osimertinib showed a significantly longer progression-free survival (PFS) and overall survival (OS) (18.9 vs 10.2 and 38.6 vs 31.8 months, respectively).^8^

The central nervous system (CNS) is the initial metastatic site in 33% of patients responding to first- and second-generation EGFR-TKIs.^9^ 21% of these metastases are identified as EGFR mutant at diagnosis.^10^ First- and second-generation TKIs are ineffective in these scenarios because their biopharmaceutical properties prevent effective penetration of the blood–brain barrier (BBB), as reflected by their low CSF concentrations compared to serum concentrations.^11,12^ Further evidence for ineffective BBB penetration is the lower frequency of Thr790Met mutations in brain metastases compared to systemic lesions after EGFR-TKI failure.^13^ In the absence of BBB penetrating drugs, the SoC is whole brain radiotherapy (WBRT), which can cause long-term cognitive impairment.^14^ Osimertinib shows greater BBB penetration and improved CNS response potential than gefitinib, rociletinib, or afatinib in animal models.^15^ Taken together, osimertinib may defer the need for brain irradiation and its detrimental toxicities.^15^ Furthermore, osimertinib has a lower half-maximal inhibitory concentration for mutant EGFR in comparison to erlotinib, gefitinib, or afatinib (0.01 vs 0.04, 0.04, and 0.08 µm, respectively).^16,17^

As previously reported, intracranial activity of osimertinib in treatment-naive and Thr790Met-positive patients produces an increased CNS ORR and PFS compared to chemotherapy (AURA3) or SoC EGFR-TKIs (FLAURA).^6,18–20^ However, none of these trials focused exclusively on osimertinib’s treatment of brain metastases nor did they assess the effect of dose escalation in the event of disease progression. High or pulsatile dosing of targeted agents in LUAD with intracranial disease was shown as beneficial.^19,21,22^ Preclinical tumor growth simulations, a documented case report, and a recent study all show that osimertinib 160 mg daily may be more effective in targeting intracranial metastases than standard dosing (80 mg).^15,23,24^

Currently, minimal studies focus on the osimertinib’s benefit in previously treated Thr790Met-negative patients.^25,26^ This phase II study evaluates the efficacy of osimertinib on treatment-naive and pretreated, EGFR-mutant, advanced LUAD patients with untreated asymptomatic brain metastases. Additionally, a novel osimertinib dose escalation regimen is tested.

## Methods

### Study Design

This nonrandomized, open-label, phase II, prospective, 3-cohort, proof-of-concept, pilot study enrolled eligible patients from Clalit Health Services in Israel and is ongoing. Eligible patients not previously treated with an EGFR-TKI were assigned to arm A. Eligible patients with documented progression on EGFR-TKI resulting in Thr790Met-positive or -negative status were assigned to arm B or C, respectively. There were no restrictions on the number of prior TKI or chemotherapy lines used in arm B or C. The primary endpoint was intracranial ORR, assessed by a neuroradiologist, defined as the proportion of patients obtaining objective complete or partial intracranial response per Modified Response Evaluation Criteria in Solid Tumors (mRECIST) criteria. Secondary endpoints were systemic ORR, intracranial and systemic disease control rate (DCR) and PFS, OS, and safety. Outcomes were calculated for each arm and separated by dose (80 vs 160 mg).

### Ethics

This trial was conducted in accordance with the Declaration of Helsinki and approved by Rabin Medical Center (approval no. 0785-15 RMC) and Soroka University Medical Center (approval no. 0299-17-SOR) ethics committees. Written informed consent was obtained at enrollment. Trial protocol is found at ClinicalTrials.gov (NCT02736513).

### Eligibility and Exclusion Criteria

Eligible patients were (1) 18 years or older with histologically or cytologically confirmed metastatic LUAD, (2) with an asymptomatic untreated brain metastasis ≥4 mm measurable by mRECIST, version 1.1, (3) with documented EGFR-TKI Thr790Met-positive or -negative sensitizing mutation, (4) WHO performance status 0 or 1, (5) with minimum life expectancy of 12 weeks, and (6) with adequate hematologic, liver and renal function. Confirmation of EGFR sensitizing mutations and Thr790Met status was completed by CLIA-certified or locally accredited laboratories through tissue specimen or plasma circulating free DNA analysis. Patients who received brain radiation were included following a 6-month interval from last treatment. Patients were excluded if they had a history of (1) interstitial lung disease or radiation pneumonitis requiring steroids, (2) uncontrolled hypertension or diabetes, (3) cardiomyopathy, (4) risk factors for arrhythmia or QTc prolongation, (5) active bleeding diathesis, or (6) active hepatitis B, C, or HIV infection. Complete criteria provided at ClinicalTrials.gov (NCT02736513).

### Treatment and Assessment Procedures

All patients were administered oral osimertinib 80 mg daily. Patients with isolated objective CNS progression were dose escalated to 160 mg of osimertinib daily. Osimertinib was held in patients with progressing intracranial disease and stereotactic radiosurgery (SRS) or WBRT was started. Following irradiation, osimertinib 160 mg was reinitiated in the absence of symptomatic systemic progression. Treatment was discontinued in those with (1) symptomatic systemic progression, (2) intracranial progression following irradiation, (3) severe toxicity, (4) study termination or withdrawal, or (5) death. Treatment was interrupted or dose reduced in select adverse events. Patients were followed for up to 5 years after first dose or death.

Modified RECIST (version 1.1) criteria assessed the intracranial tumor response by identifying CNS target lesion ≥4 mm.^27,28^ Brain gadolinium-enhanced MRI was taken at baseline, every 6 weeks during the first 3 months, and every 3 months thereafter. Standard RECIST (version 1.1) assessed systemic efficacy through positron emission tomography–computed tomography preformed at baseline and every 3 months.

Systemic and intracranial endpoints were evaluated using RECIST and mRECIST, respectively. Systemic ORR is the proportion of patients achieving systemic complete response (CR) or partial response (PR). DCR is the percentage achieving best overall response (systemically or intracranially) consisting of patients with CR, PR, or stable disease. PFS is the time from initial dose until objective disease progression or death, systemically or intracranially. OS is the time from drug initiation to death from any cause.

Adverse events were graded via the Common Terminology Criteria for Adverse Events (CTCAE), version 4 and monitored for 28 days following discontinuation of osimertinib. Further details on monitored measures can be found at ClinicalTrials.gov (NCT02736513).

### Sample Size Calculation and Statistical Analysis

We hypothesized that osimertinib is an effective targeted agent, active in the CNS of EGFR-mutant patients with or without Thr790Met mutation. Therefore, we expected an intracranial ORR of ≥30%, while an ORR of <10% was considered failure of drug activity. Since this is a pilot study, we planned to enroll 20 patients in each arm. At the data cutoff point, July 31, 2020, 48 patients with asymptomatic brain metastases started osimertinib treatment (arm A = 20, arm B = 18, arm C = 10). The statistical analysis of the PFS and OS was analyzed by a Kaplan–Meier test with log-rank *P* value. The intra-arm analysis of the number and diameter of target lesions used the nonparametric Wilcoxon signed ranks test. The interarm analysis utilized the nonparametric Mann–Whitney test. *P* < .05 indicated statistical significance. Statistical analysis was conducted with SPSS build 1.0.0.1508 and RStudio Version 1.3.1073.

## Results

Between May 31, 2016 and July 31, 2020 (data cutoff point), 48 patients with asymptomatic brain metastases were enrolled (20 in arm A, 18 in arm B, and 10 in arm C). One patient from arm A withdrew within 2 weeks, after molecular profiling confirmed an ALK mutation and EGFR-negative status; this patient was excluded from the analysis. Patient demographics and brain lesion characteristics are shown in Table 1.

**Table 1.** Patient Demographics and Brain Lesion Characteristics

Characteristics	No. (%)		
	Arm A (Treatment Naive, *n* = 19)	Arm B (Pretreated, Thr790Met Positive, *n* = 18)	Arm C (Pretreated, Thr790Met Negative, *n* = 10)
Age			
Years (range)	66 (31–78)	66 (63–80)	60 (48–71)
Sex			
Male	5 (26)	9 (50)	3 (30)
Female	14 (73)	9 (50)	7 (70)
Smoking status			
Never	15 (78)	17 (95)	6 (60)
Past	4 (21)	1 (5)	4 (40)
WHO performance status			
1	7 (36)	12 (66)	8 (80)
0	12 (63)	6 (33)	2 (20)
Baseline EGFR mutation^a^			
Ex 19 DEL	12 (63)	8 (43)	8 (80)
L858R	6 (31)	10 (57)	2 (20)
S768I	0 (0)	2 (11)	0 (0)
L861Q	0 (0)	1 (5)	0 (0)
delE709_T710insD	1 (5)	0 (0)	0 (0)
G719A	0 (0)	1 (5)	0 (0)
Thr790Met	0 (0)	18 (100)	0 (0)
Histology			
Adenocarcinoma	19 (100)	18 (100)	10 (100)
Prior EGFR-TKI treatment			
Erlotinib	0 (0)	2 (11)	2 (20)
Gefitinib	0 (0)	4 (22)	2 (20)
Afatinib	0 (0)	12 (66)	6 (60)
Prior systemic response to EGFR-TKI therapy			
PR	NA	0 (0)	0 (0)
SD	NA	4 (22)	3 (30)
PD	NA	14 (77)	7 (70)
Prior CNS therapy^b^			
Surgical resection	0 (0)	1 (5)	1 (10)
WBRT	0 (0)	1 (5)	1 (10)
SRS	0 (0)	1 (5)	0 (0)
Brain Metastases	Median No. (Range)		
Targeted brain lesions	3.47 (1–11)	3.1 (1–9)	2.44 (1–5)
Diameter of targeted brain lesions, mm	31.3 (4–90.8)	23.68 (4–52.5)	28.15 (7.6–57.9)

CNS, central nervous system; Ex 19 DEL, exon 19 deletion; NA, not applicable; PD, progressive disease; PR, partial response; SD, stable disease; SRS, stereotactic radiosurgery; TKI, tyrosine-kinase inhibitor; WBRT, whole brain radiotherapy; WHO, World Health Organization.

^a^The sum of counts for baseline *EGFR* mutations in arm B differs from the number of patients in arm B because of 2 patients each harboring a compound *EGFR* mutation of L861Q + G719A.

^b^The sum of counts for previous EGFR-TKI treatments in arm B differs from the number of patients in arm B because 1 patient was treated with both gefitinib and afatinib.

^c^All previous CNS therapies were performed on nontarget lesions with the exception of 1 patient from arm B treated with stereotactic radiosurgery more than 6 months prior to osimertinib treatment.

Most target lesions were untreated, with the following exceptions: from arm B, 1 patient underwent surgical resection, 1 was treated with WBRT, and 1 underwent SRS; in arm C, 1 patient underwent surgical resection and another received WBRT. At the data cutoff point, all participants were evaluated for both brain and systemic responses.

**Figure 1.**
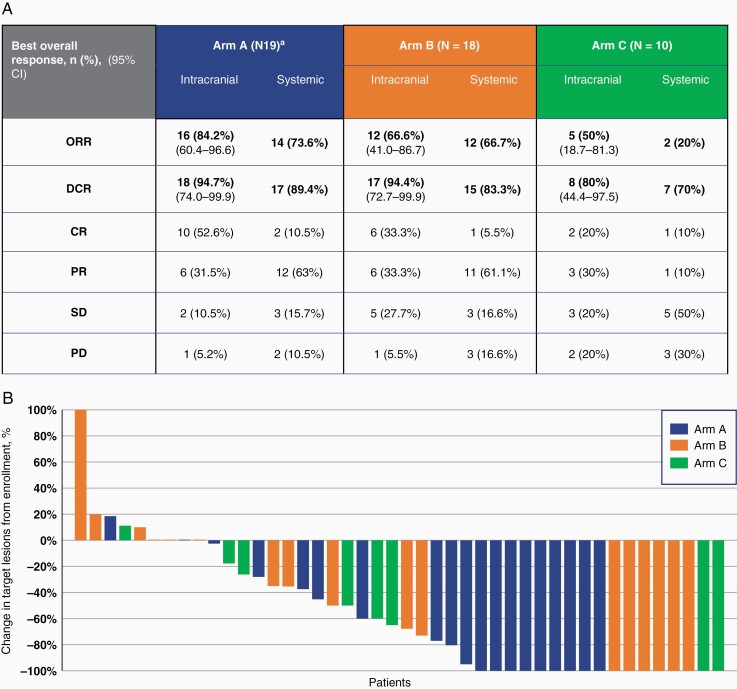
Efficacy of osimertinib in all treatment arms. (A) Illustrates the intracranial and systemic efficacy of osimertinib in each arm of the trial. The percent of patients that reached ORR in each study arm is represented by each bar. 95% CIs for intracranial ORR and DCR are included in blocked parentheses. ^a^Analysis was done on 19 patients with 1 participant being excluded following molecular profiling. CR, complete response; DCR, disease control rate; ORR, objective response rate; PD, progressive disease; PR, partial response; SD, stable disease. (B) Waterfall plot of each arm demonstrates the best percentage change in intracranial target lesions from baseline. Complete response (*n* = 18), partial response (*n* = 15), stable disease (*n* = 10), and progressive disease (*n* = 4).

In arm A, the intracranial ORR (80 mg) was 84.2% (*n* = 16/19) [95% CI 60.4–96.6], while intracranial DCR was 94.7% (*n* = 18/19) [95% CI 74.0–99.9]. There were 10 patients with CR, 6 with PR, and 2 with stable disease. In arm B, the intracranial ORR was 66.7% (*n* = 12/18) [95% CI 41.0–86.7], while intracranial DCR was 94.4% (*n* = 17/18) [95% CI 72.7–99.9]. There were 6 patients with CRs, 6 with PRs, and 5 with stable disease. In arm C, the intracranial ORR was 50% (*n* = 5/10) [95% CI 18.7–81.3], while intracranial DCR was 80% (*n* = 8/10) [95% CI 44.4–97.5]. There were 2 patients with CRs, 3 with PRs, and 3 with stable disease (Figure 1A and B).

**Figure 2.**
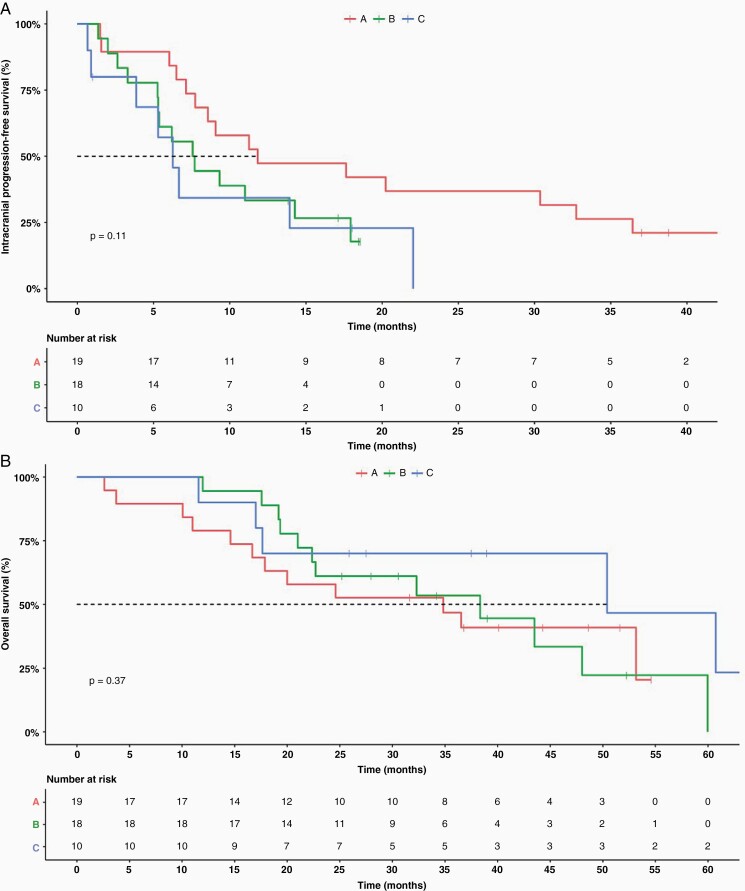
(A) The Kaplan–Meier plot of intracranial progression-free survival (iPFS) by study arm (*P* = .11), the dashed line indicates 50% (median iPFS). (B) The Kaplan–Meier plot of overall survival (OS) by study arm (*P* = .37), the dashed line indicates 50% (median OS).

The median intracranial PFS (iPFS) was 11.8 months in arm A [95% CI 7.73 to NA] with a range of 1.5–43.1 months, 7.6 months in arm B [95% CI 5.30 to NA] (range 1.4–18.6 months), and 6.3 months in arm C [95% CI 3.9 to NA] (range 0.7–22 months) (Figure 2A). The median systemic PFS for arms A, B, and C was 18.5, 9.3, and 7.9 months, respectively (arm A: [95% CI 1.5–43.1]; arm B: [95% CI 1.4–18.6]; arm C: [95% CI 0.7–22]). The median OS was 34.8 months in arm A [95% CI 17.9 to NA], 38.3 months in arm B [95% CI 22.4 to NA], and 50.4 months in arm C [95% CI 17.6 to NA] (Figure 2B). The median follow-up in arm A was 32.1 months with a range of 0.1–57.8 months, while arm B had a median of 13.7 months, ranging from 2.6 to 34.2 months, moreover, arm C had a median follow-up of 12.2 months with a range of 0.9–23.4 months.

**Figure 3.**
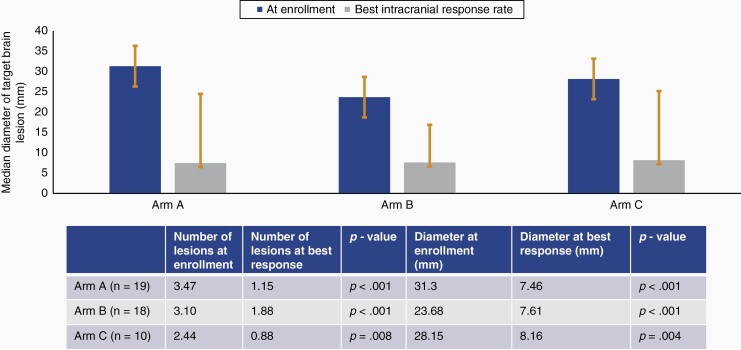
Illustrates the changes in diameter of target brain lesions from the time of enrollment to the time of best intracranial response. A significant decrease in the median diameter of target brain lesions is seen throughout all arms. The number and diameter of target lesions, and their corresponding *P* values, at each time point, are proved in the table.

**Figure 4:**
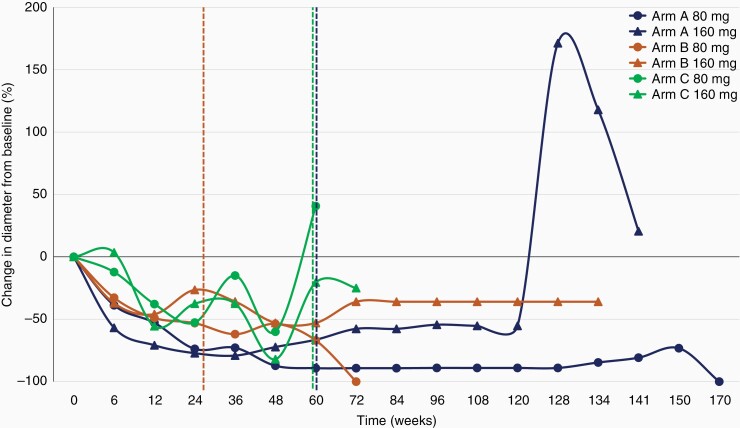
Spider chart illustrating the median change in diameter from baseline for each arm. Dashed vertical lines represent the median time on trial to dose escalation for arm A = 61.33 weeks (blue), arm B = 28.5 weeks (orange), and arm C = 60 weeks (green).

As part of intracranial RECIST criteria, we assessed the number and diameter of target lesions, once at enrollment, and again at the time of the best intracranial response. Throughout all arms, there was a significant decrease in both parameters. In arm A, the median number of target brain lesions decreased significantly from 3.5 to 1.2 (*Z* = 3.207; *P* < .001). The median diameter significantly decreased from 31.3 to 7.46 mm (*Z* = 3.823; *P* < .001). In arm B, both the median number of target lesions and their median diameter significantly reduced from 3.1 to 1.3 (*Z* = 2.949; *P* < .001) and from 23.7 to 7.6 mm (*Z* = 3.408; *P* < .001). In arm C, too, these measures significantly decreased, from 2.4 to 0.9 (*Z* = 2.640; *P* = .008) and from 28.2 to 8.2 mm (Z = 2.668; *P* = .004), respectively (Figure 3). Furthermore, there was no difference between arms B and C when evaluating the number and diameter of target lesions at the time of best intracranial response (lesions: *U* = 66.0, *P* = .463; diameter: *U* = 68, *P* = .527). The changes in diameter of target brain lesions in each participant, throughout the trial, are shown in Figure 4.

We previously reported on 11 patients on the dose escalation protocol (arm A = 5, arm B = 4, arm C = 2).^29^ The pooled intracranial response rate to dose escalation was 54%, with DCR of 72.7%. The median intracranial PFS was 4.3 months; 3.8 ± 6.4 (1.8–18.9), 5.6 ± 9.7 (0.7–25.5), and 7.0 ± 2.7 (4.3–9.6) for arms A, B, and C, respectively.^29^ Currently, there are 13 patients (arm A = 6, arm B = 5, arm C = 2) on the dose escalation protocol; these results are premature.

At the cutoff date, 20%, 11%, and 50% of patients in arms A, B, and C, respectively, continued responding to osimertinib 80 mg (see Supplementary Figure S1, which show arm allocations). The adverse events are listed in Table 2. Overall, they were mild, without any serious presentations.

**Table 2.** Adverse Events (AEs) in All Study Participants

AE	No. (%) Grades 1 and 2	No. (%) Grade 3	No. (%) Grade 4
Fatigue	16 (33%)	2 (4%)	0
Nail toxicity	12 (25%)	2 (4%)	0
Rash	11 (22%)	2 (4%)	0
Dry skin	11 (22%)	0	0
Decrease appetite	10 (20%)	0	0
Diarrhea	8 (16%)	1 (2%)	0
Headache	7 (14%)	0	0
Pruritis	6 (12%)	0	0
Nausea	5 (10%)	0	0
Thrombocytopenia	4 (8%)	0	0
Leukopenia	3 (6%)	0	0
Cough	3 (6%)	0	0
Constipation	3 (6%)	0	0
Acne	3 (6%)	0	0
Anemia	2 (4%)	0	0
Stomatitis	1 (2%)	0	0

Safety analyses include all the patients enrolled in the trial. Some patients had more than one adverse event.

## Discussion

Currently, osimertinib is the standard of care in patients with advanced or metastatic EGFR-mutant LUAD. It is preferred as a first-line treatment, including for patients with acquired resistance to prior EGFR-TKIs via Thr790Met mutations. Unfortunately, EGFR-mutant NSCLC patients commonly present with brain metastases: 70% compared to 38% in EGFR wild-type patients.^30^ Osimertinib shows greater BBB penetration in clinical trials when compared with other EGFR-TKIs.^31^ In the phase III AURA3 trial (*n* = 144), osimertinib was superior to chemotherapy in treatment of CNS metastases in the pretreated Thr790Met-positive setting (*n* = 75), with higher CNS ORR (70% vs 31%) and longer CNS PFS (11·7 vs 5·6 months).^6,19^ This present study aimed to evaluate the intracranial ORR, DCR, and iPFS in patients that were either treatment naive or previously treated with EGFRThr790Met-positive or -negative mutation status. Additionally, we examined the number and diameter of brain target lesions. Finally, we assessed the intracranial ORR, DCR, and iPFS in patients that underwent dose escalation in the presence of additional brain metastases.

Evidence of activity of osimertinib in LUAD brain metastases was first presented in 2017 by Goss et al.^18^ Their study demonstrated that confirmed CNS ORR and DCR were 54% and 92%, respectively. Our data show that the median intracranial ORR was 84.2%, 66.7%, and 50% in arms A, B, and C respectively, while the intracranial DCR was 94.7%, 94.4%, and 80% in arms A, B, and C, displaying osimertinib’s activity on brain metastases. Of note, after pooling the arms together, our median intracranial ORR and median intracranial DCR are 66.93% (95% CI 50%–84%) and 89.7% (95% CI 80%–94%), respectively. These pooled findings are similar to a recently published meta-analysis that included 15 studies with 324 EGFR mutated patients, either treatment naive or previously treated: they showed a median CNS ORR of 64% (95% CI 53%–76%) and a median CNS DCR of 90% (95% CI 85%–93%).^26^

Furthermore, our results correlate with a pooled analysis of 2 phase II trials (*n* = 50) that tested second-line osimertinib in pretreated, EGFR Thr790Met-positive LUAD patients with CNS metastases.^18^ This pooled analysis showed a CNS ORR of 54% (95% CI 39–68) and a DCR of 92% (95% CI 81–98). It was not possible to calculate the iPFS for these studies (95% CI 7 to not calculable).^18^ Participants in arm B of our trial are similar to those studied in the pooled analysis; the iPFS of these patients is 7.6 months (95% CI 5.30 to NA).

In arm A of our study, the median intracranial response was 84.2% while the iPFS was 11.8 months (95% CI 7.7 to NA). The phase III FLAURA trial reports a median iPFS of 15.2 month (95% CI 12.1–21.4) in 53 patients with CNS metastases being treated with upfront osimertinib. These results support our findings and indicate that intracranial activity of upfront osimertinib is superior to first- or second-generation EGFR-TKIs, presenting a survival benefit.^20^

Interestingly, the median iPFS of patients in arm C is 6.3 months (95% CI 3.9 to NA). This is similar, although slightly higher, than 5.1 months, which was the PFS observed by the TREM study in patients that were EGFR Thr790Met negative. However, unlike our study, the TREM study did not focus specifically on iPFS but on PFS in general, which may account for the slight difference. Important to note, 20% (*n* = 2/10) of patients in arm C of our study obtained CR, while only 2% (*n* = 1/50) of similar patients in the TREM study showed CR.^25^

There was a significant decrease in the number of target lesions and their diameters at the time of best intracranial response, irrespective of Thr790Met mutation status (lesions: arm A, *P* < .001; arm B, *P* < .001; arm C, *P* = .008) (diameter: arm A, *P* < .001; arm B, *P* < .001; arm C, *P* = .004). Furthermore, there was no difference between arms B and C regarding these parameters (target lesions: *U* = 66.0, *P* = .463; diameter: *U* = 68, *P* = .527). This finding suggests that osimertinib should be the preferred treatment option in EGFR patients with brain involvement, regardless of their Thr790Met mutation status.

In patients with isolated brain progression on osimertinib 80 mg, dose escalation to 160 mg QD yielded intracranial response rate of 54%, and the pooled DCR rose to 72.7%, as we previously published.^29^

For many years, the cornerstone of management of multiple brain metastases was WBRT. Recently, multiple rounds of SRS were also accepted as an efficient and less toxic treatment strategy for patients with up to 10 brain lesions.^32^ Yet, patients with EGFR-mutant LUAD may already have numerous brain lesions very early in their disease.^33^ Previous report have shown that 65% of the EGFR-positive patients have brain metastasis at presentation, in comparison with 35% of EGFR-negative patients.^34^ This study indicates that osimertinib used as first-line therapy is highly efficient and may postpone, and even avoid, the need for WBRT and its deleterious long-term complications. Deferring SRS is also advantageous, as SRS long-term complications include necrosis and secondary edema, particularly when the radiated lesion is bigger than 1.6 cm.^35^ Our results showed clear intracranial response in both arms B and C, with significant reduction in the number and diameter of targeted brain lesions. These findings further demonstrate that osimertinib should be considered the primary treatment option in EGFR patients with isolated brain metastases, regardless of their Thr790Met mutation status.

Key limitations of this study are the short follow-up duration and small number of participants. These factors may impact the response rates and survival outcomes, yet our results are very similar to the values previously reported in the literature. It is important to note that arm A was limited to participants with EGFR exon 19 deletions and L858R mutations, representing 86% of the EGFR-mutant forms in LUAD.^36^ Although this may skew the results in favor of osimertinib, the high intracranial response rates seen throughout all studied arms suggests that osimertinib can be considered by clinicians that are aiming to spare or delay brain radiotherapy, irrespective of the patients EGFR mutation status. Future large-scale prospective studies are needed to further elucidate osimertinib’s effectiveness on other EGFR-mutant forms. The phase I BLOOM trial reported that 33% (*n* = 7/21) of pretreated, EGFR-mutant, advanced LUAD patients with leptomeningeal metastases responded to osimertinib 160 mg daily and achieved an intracranial DCR of 76% (*n* = 16/21).^37,38^ Only 2 patients with leptomeningeal disease were included in this present trial. Future trials should evaluate osimertinib’s efficacy and safety in patients with leptomeningeal disease, as well as the potential for dose escalation.

To conclude, analysis of this phase II trial indicates that osimertinib is highly effective for brain metastasis in naive EGFR-mutant LUAD, and as second line treatment in both EGFR Thr790Met-positive and -negative patients. Even in the presence of Thr790Met mutations, osimertinib showed high intracranial efficacy. Notably, there was a significant decrease in the number and diameter of target brain metastases in all arms of the study. Therefore, we suggest consideration of osimertinib as a treatment option in EGFR-mutant patients with brain involvement, regardless of their Thr790Met mutation status.

## Supplementary Material

Supplementary material is available at *Neuro-Oncology Advances* online.

Supplementary Figure S1. Participant allocation to the different study arms. EGFRm, epithelial growth factor receptor mutated.

vdab188_suppl_Supplementary_Figure_S1
