# Supplementary figures and images for: Osimertinib in advanced EGFR-mutant lung adenocarcinoma with asymptomatic brain metastases: an open-label, 3-arm, phase II pilot study
Source: Neurooncol Adv. 2021 Dec 27;4(1):vdab188. doi: 10.1093/noajnl/vdab188 (PMC8826702; doi:10.1093/noajnl/vdab188)

## Slide 1
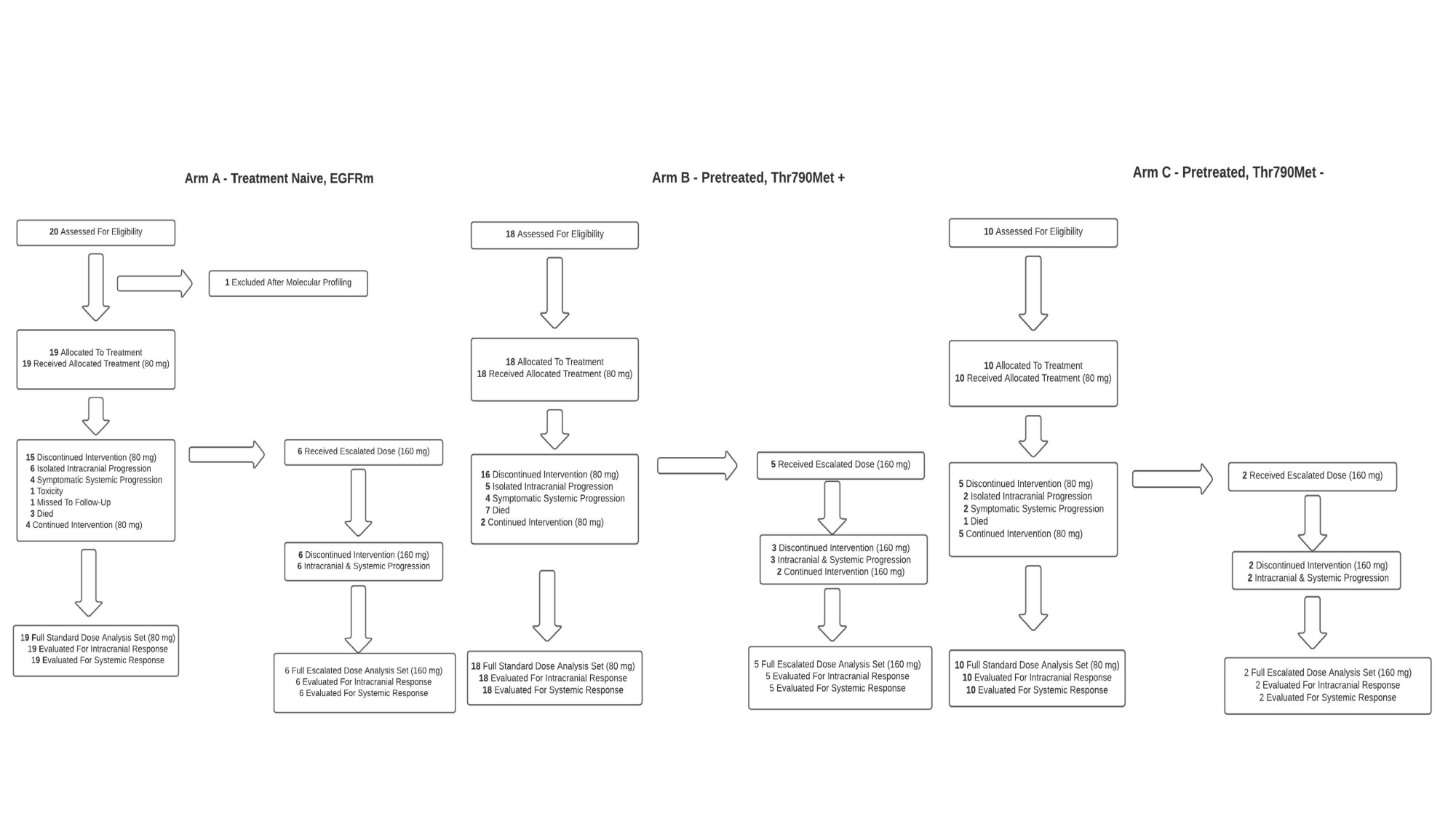

Supplement: vdab188_suppl_Supplementary_Figure_S1 [file vdab188_suppl_supplementary_figure_s1.pptx]
